# The effectiveness of social bubbles as part of a Covid-19 lockdown exit strategy, a modelling study

**DOI:** 10.12688/wellcomeopenres.16164.2

**Published:** 2021-03-29

**Authors:** Trystan Leng, Connor White, Joe Hilton, Adam Kucharski, Lorenzo Pellis, Helena Stage, Nicholas G. Davies, Centre for Mathematical Modelling of Infectious Disease 2019 nCoV Working Group, Matt J. Keeling, Stefan Flasche

**Affiliations:** 1 The Zeeman Institute for Systems Biology & Infectious Disease Epidemiology Research, University of Warwick, Coventry, UK; 2 Centre for Mathematical Modelling of Infectious Diseases, London School of Hygiene & Tropical Medicine, London, UK; 3 Department of Mathematics, University of Manchester, Manchester, UK

**Keywords:** Contact clustering, Social bubble, Exit strategy, Covid-19

## Abstract

**Background:** *​ *During the coronavirus disease 2019 (COVID-19) lockdown, contact clustering in social bubbles may allow extending contacts beyond the household at minimal additional risk and hence has been considered as part of modified lockdown policy or a gradual lockdown exit strategy. We estimated the impact of such strategies on epidemic and mortality risk using the UK as a case study.

**Methods:** *​ *We used an individual based model for a synthetic population similar to the UK, stratified into transmission risks from the community, within the household and from other households in the same social bubble. The base case considers a situation where non-essential shops and schools are closed, the secondary household attack rate is 20% and the initial reproduction number is 0.8. We simulate social bubble strategies (where two households form an exclusive pair) for households including children, for single occupancy households, and for all households. We test the sensitivity of results to a range of alternative model assumptions and parameters.

**Results:**  Clustering contacts outside the household into exclusive bubbles is an effective strategy of increasing contacts while limiting the associated increase in epidemic risk. In the base case, social bubbles reduced fatalities by 42% compared to an unclustered increase of contacts. We find that if all households were to form social bubbles the reproduction number would likely increase to above the epidemic threshold of R=1. Strategies allowing households with young children or single occupancy households to form social bubbles increased the reproduction number by less than 11%. The corresponding increase in mortality is proportional to the increase in the epidemic risk but is focussed in older adults irrespective of inclusion in social bubbles.

**Conclusions: ​** If managed appropriately, social bubbles can be an effective way of extending contacts beyond the household while limiting the increase in epidemic risk.

## Background

In the UK, similar to many other countries, the introduction of stringent physical distancing measures in March 2020 in response to the coronavirus disease 2018 (COVID-19) pandemic has reduced the transmission of severe acute respirator syndome coronavirus 2 (SARS-CoV-2) and alleviated the burden on the healthcare system ^1, 2^. However, this reduction has come at great economic, societal, and wider health costs ^3– 6^. As infection incidence has declined, countries have begun to ease restrictions in an attempt to reduce the societal and economic burden of lockdown. However, with infection incidence beginning to rise again in some countries, and with the spectre of a potential second epidemic wave in the Winter of 2020, countries must now strike a balance between easing restrictions and ensuring that the epidemic remains under control ^7– 11^.

Multiple options, that could in combination form an exit strategy, have been proposed to allow easing of restrictions. These include: the widespread use of rapid, potentially app-based, contact tracing in combination with rapid testing and self-isolation ^12, 13^; expanded random testing to increase detection of asymptomatic infection ^14– 16^; strict quarantining of travellers on arrival ^17, 18^; and the use of face masks in high-risk environments ^19– 22^. Another potential component of a lockdown exit strategy that could allow for greater social interaction is the clustering of contacts beyond the household, commonly referred to as the social bubble or the ‘double bubble’ strategy ^23– 27^. Under this strategy, households are allowed to form a cohesive unit with one other household, generating a ‘social bubble’; this allows individuals to increase their close, physical social interactions beyond their household while potentially limiting the risk of infection through the exclusivity of the bubble. A similar strategy has been implemented in some countries, including New Zealand and Germany, and is currently part of the lockdown exit strategy in the UK ^28^. 

While physical distancing has placed additional pressures on society as a whole, some households are likely to be disproportionately more at risk of social isolation. Many adults in the UK will have been able to partially shift social contacts online and since 11th May (and 1st June) have been allowed to socialise outdoors with a maximum of one (and subsequently up to five) others while adhering to distancing guidelines ^29^. However, such social contact replacements can be more difficult for children, for whom verbal interaction is only a small part of their communication with peers. Further, their carers have often had to balance working from home, childcare and homeschooling, generally without being able to access a support network from family, friends or professional childminders ^30^. Single occupancy and single parent households have also likely been disproportionately affected as the complete absence of social face-to-face interactions for many months may impact mental wellbeing ^31, 32^. To address this, households with one adult have been allowed to form a ‘support bubble’ with another household in England and Northern Ireland since 13th June ^33^. 

Here, using mathematical models, we assess the likely increase in transmission generated by various plausible social bubble strategies and use the UK as a case study. In particular, we compare the impact of limiting bubbles to those households who would benefit most (single occupancy households and those with young children) with allowing all households to form bubbles. We assess these changes in terms of both the increase in transmission (as characterised by the reproductive ratio, *R*) and short-term increase in fatalities.

## Methods

### Population

**Figure 1.**
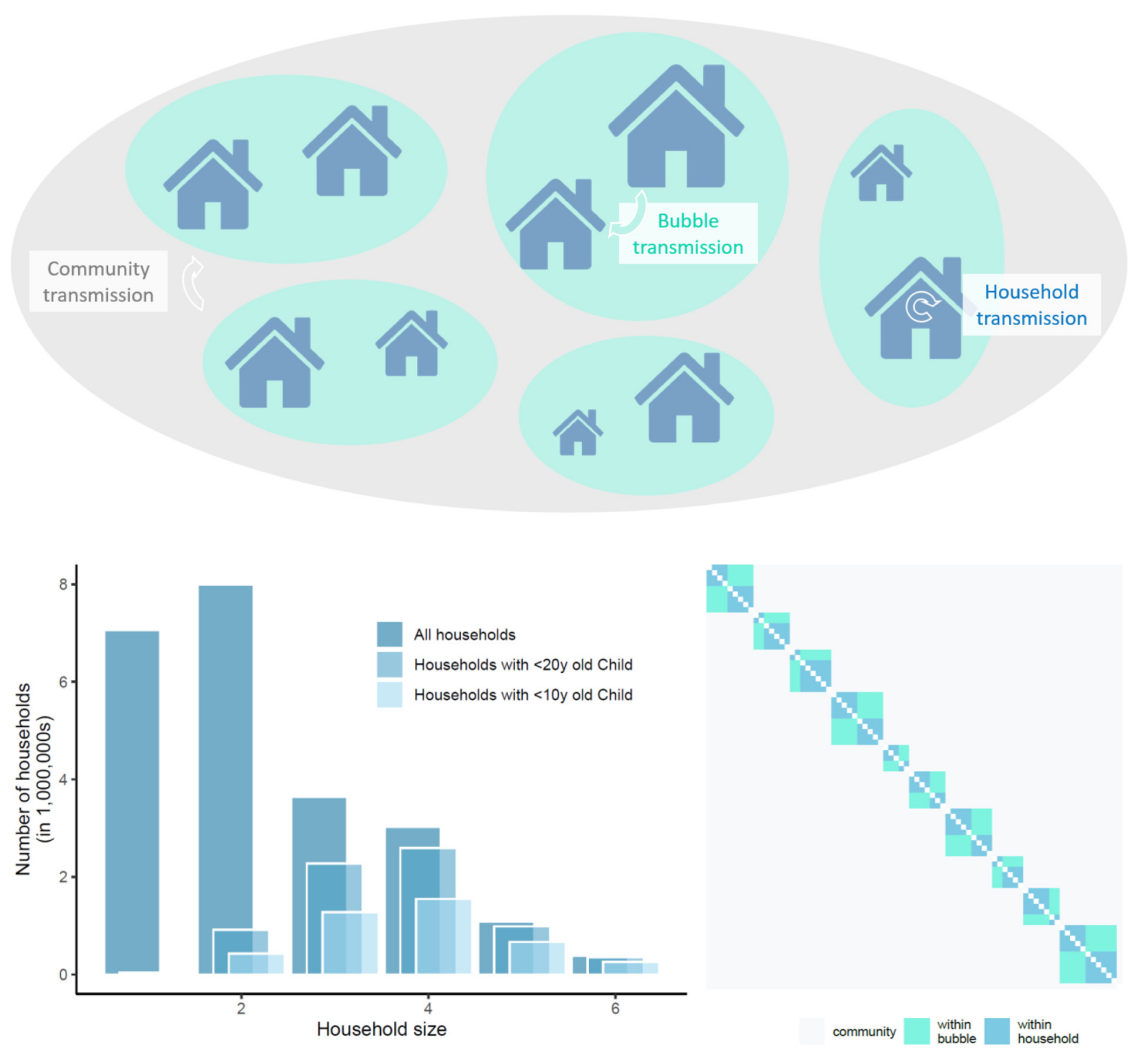
top panel: schematic of model structure and its stratification into different household sizes with three components of transmission dynamics, community transmission, bubble transmission and household transmission; left panel: household size distribution for all households in England and Wales, for those households with at least one child younger than 20-years-old and for those with at least one child younger than 10-years-old (about primary school age and younger). Right panel: illustrative transmission probability matrix *A*, composed of household and bubble contacts and including community transmission.

The model’s synthetic population was created by generating individuals who are residents of one of 10,000 households. The size of the individual households, as well as the age distribution within households, was sampled to match that observed in the most recent census in England and Wales in 2011 ( Figure 1) ^34^.

We used data from the 2011 census of England and Wales to construct a distribution of age-stratified household compositions in terms of 10-year age bands. Each household composition consisted of the number of individuals in each age band belonging to the household. We assigned probabilities to each composition observed in the census data based on the frequency of its appearance, and then used these probabilities to construct our simulated household population. This gave us a synthetic population whose age structure was comparable with that of England and Wales and whose household compositions reflected the observed correlations between the ages of household occupants. In particular, this formulation should realistically capture the generational structure of households in England and Wales, which we expect to be an important factor in transmission across age classes.

### Transmission model

The transmission dynamics are set to simulate the status of COVID-19 interventions during ‘lockdown’ in the UK in May 2020 or future lockdowns to mitigate a second wave, in particular simulating contacts that are substantially reduced and largely household-based, with schools, non-essential retail, and leisure facilities closed. This is achieved through stochastic simulation of infections spreading through an interconnected population by generation; connections are captured by a matrix, *A,* which defines the probability that infection can pass between any two individuals in the population over an individual’s entire infectious period. *A* is composed of the sum of two matrices *H* and *B*, which capture within-household and within-bubble transmission respectively ( Figure 1). We can then use the matrix *A* to drive forwards the stochastic dynamics using a next generation approach. To this, we add random (mean-field) transmission between individuals in the population to simulate the risk that infection in the wider community poses to the household and the social bubble, and vice versa. Transmission rates within the household and the wider community are matched to observed data on secondary attack rates and population-scale *R* estimates. We assume that households are adhering to current restrictions and social distancing, and therefore largely act as a coherent and largely isolated unit. We therefore assume that the risk of a household acquiring infection from the community is independent of its number of occupants as observed in a cross-sectional serological study for SARS-CoV-2 in Germany in March and April 2020 ^35^.

We assume that susceptibility to infection as well as transmissibility of infection can be age dependent, hence transmission rates across contacts depend on the age of both individuals.. There are two conflicting bodies of evidence about the potential role of children. Firstly, it has been observed that children are more likely to experience mild or no symptoms, and as such may have a lower transmission rate ^36, 37^. Secondly, cases with more severe symptoms are likely to self-isolate reducing their effective infectious period, therefore children that are asymptomatic (or mildly symptomatic) may continue to transmit for longer ^35^. In our base parameterisation, we assume that children are 50% as susceptible to infection as adults or elderly adults, but assume that transmissibility is independent of age; this echoes the assumptions of a previous model ^9^, but an alternative parameterisation based on other work ^8^ is considered as part of sensitivity analysis. We assume that transmission across close contacts (household or bubble) depends on the interaction between two individuals, and that the amount of ‘interaction’ an individual has with a close contact is inversely proportional to the number of that type of close contacts that person has ^35, 38, 39^. Under this assumption, transmission within households and across households who share a bubble is frequency dependent. 

Throughout, we compare the baseline model without the additional social interactions via bubbles (C1) with different ways in which bubbles could be allowed to form (scenarios 1–6, see below). To assess the effectiveness of social bubbles, compared to increasing contacts in an unclustered fashion, we consider two further comparison scenarios. In C2, individuals make the same number of infectious contacts with the population as in Scenario 6, but these are chosen randomly across the population. In C3, individuals’ infectious contacts are resampled every generation. Scenario 6, C2, and C3 therefore represent fixed and clustered, fixed and unclustered, and variable additional contacts respectively.

### Technical model summary

In this subsection we describe the model and its underlying assumptions in detail. Table 1 describes our notation, while Table 2 contains the formulae for transmission rates within our model. Key model parameters and assumptions are described below in Table 3.

**Table 1.** Model notation.

Symbol	Meaning
*C(i)*	Age-dependent susceptibility scaling factor of individual *i*
*T(i)*	Age-dependent transmissibility scaling factor of individual *i*
*N _H_(i)*	Number of individuals in an individual *i*’s household
*τ _H_*	Baseline transmission rate across household contact
*ρ _H_(i,j)*	Household transmission rate from individual *j* to individual *i*
*τ _B_*	Baseline transmission rate across bubble contact, *τ _B_* = kτ *_H_* where k ∈ [0 1]
*ρ _B_(i,j)*	Bubble transmission rate from individual *j* to individual *i*
*ε*	Baseline mean-field transmission rate
*ε(i)*	Mean-field transmission rate to an individual *i*
*I _g_(i)*	Infection status of an individual *i* at generation *g*. *I _g_(i)* = 0 if *i* is susceptible at generation *g*. *I _g_(i)* = 1 if *i* has been infected by generation *g* (so includes recovered individuals)
*S*_g_ *(i)*	1 - *I _g_(i)*, i.e. the susceptibility status of an individual *i* at generation *g*.
*N*	Size of population.

**Table 2.** Transmission rates.

Transmission rate	Model formulation
Household, *ρ _H_(i,j)*	$\frac{2T(j)c(i)\tau_{H}}{N_{H}(i)-1}$ if *i* and *j* are within the same household, 0 otherwise
Bubble, *ρ _B_(i,j)*	$T(j)C(i)(\frac{\;\tau_{B}}{N_{H}(i)}+\frac{\;\tau_{B}}{N_{H}(j)})$ if *i* and *j* are within the same bubble (but not the same household), 0 otherwise
Mean-field, *ε(i)*	$\frac{C(i)\epsilon }{N_{H}(i)}\frac{\sum_{j\;}\;T(j)(I_{g}(j)-I_{g-1}(j))N_{H}^{-1}(j)}{\sum_{j}\;N_{H}^{-1}(j)\;}$

**Table 3.** Key model parameters and assumptions.

Parameter	Description	Value (base case)	Value (Sensitivity)	Source
	Household structure and age distribution			34
*τ _H_*	50% of the transmission rate for an adult, in a two person household	0.345 (20% SAR)	0.155 (10% SAR) 0.86 (40% SAR)	Calibrated to 40, 41
*τ _B_*	Transmission rate for an adult within the bubble	0.5 *τ _H_*	1 *τ _H_* 0.1 *τ _H_*	*assumption*
	Relative transmissibility of a child and older adult vs adults	1 and 1	0.64 and 2.9	8, 9
	Relative susceptibility of a child and older adult vs adults	0.5 and 1	0.79 and 1.25	8, 9
	Infection fatality rate	In 10y age bands		42
*R _e_*	Net reproduction number	0.8	0.7, 0,9	43
*ε*	Rate of infection from the community	1.13 (20% SAR)	1.29 (10% SAR) 0.925 (40% SAR)	Calibrated to *R _e_ given τ _H_*

The expressions for household and bubble transmission rates are derived by assuming that transmission between close contacts depends on frequency of interaction between those individuals. We decompose interaction between individuals *i* and *j* into interaction led by *i* and interaction led by *j*. The amount of interaction led by *i* (or *j*) ** depends on the number of other close contacts *i* (or *j*) ** has. The total amount of interaction, hence the rate of transmission, is given by summing these interactions. For household contacts *i* and *j*, both individuals have the same number of household contacts, hence leading to the above formula, equivalent to the standard frequency dependence of transmission assumption. Across bubble contacts, interaction depends on the size of both households. To obtain equations for density-dependent transmission, the dividing N _H_ terms are omitted from the equations for *ρ _H_(i,j)* and *ρ _B_(i,j)*.

Specific transmission rates also depend upon the transmissibility of the individual *j* transmitting infection ( *T(j)*), and upon the susceptibility of the individual *i* receiving infection ( *C(i)*), which are dependent on the age classes of individuals *j* and *i*.

The mean-field transmission to an individual, as well as an individual’s contribution to mean-field infection, is inversely proportional to the number of individuals in their household, as we assume that a household acts as a coherent and largely self-contained unit when interacting with the population at large. *ε(i)* also depends on the susceptibility of *i*, determined by their age class. The force of infection from the general population is given by $\sum_{j\;}\;T(j)(I_{g}(j)-I_{g-1}(j))N_{H}^{-1}(j),$ i.e. the new infections in generation *g*, scaled by both the relative transmissibility of newly infected individuals and by their interaction with the general population determined by their household size.

By considering transmission as a Poisson process, we obtain the elements of the probability matrices *H* and *B*, the matrices of within household and within bubble transmissions respectively, by taking *H*( *i*, *j*) = 1-e ^-⍴H(i,j) ^ and *B*( *i*, *j*) = 1-e ^-⍴B(i,j)^. A non-zero element within the matrix *H* (or *B*) indicates that the corresponding individuals are within the same household (or bubble). We obtain the overall probability matrix for the population by taking *A* = *H*+ *B*.

In order to simulate an epidemic, we begin by randomly sampling the probability matrix *A*. Doing so, we retain only the infectious connections between individuals that will lead to an infection. These sampled probability matrices therefore represent *potential* transmission networks (though the exact transmission network for a given simulation will depend upon who is initially infected). Because these sampled matrices refer to transmission events rather than contacts, this matrix may be unsymmetric. We refer to the sampled matrix as *A*’. *A*’( *i*, *j*) = 1 denotes that individual *j* will infect individual *i* with probability 1, given individual *j* is infected. We initiate each simulation with the required number of infectious individuals for 1% of the population to be infected by generation 4. Initially infected individuals are chosen with probability proportional to their mean-field interaction, i.e. inversely proportional to their household size. Letting **I _g_** be the vector of infection statuses of individuals in generation *g*, we obtain the next generation by **I _g+1_** = sign(( *A*’+ *Id*) X **I _g_**), where *Id* is the identity matrix, and where sign() is an element-wise function equal to 1 for each positive element and 0 otherwise. Via this matrix multiplication, every newly infected individual in generation *g* infects all of their infectious contacts that generation. Here the identity matrix is added to impose that individuals do not become susceptible again after one generation, while the sign function is used to impose that individuals cannot be infected more than once. This process can be iterated until equilibrium is reached, and the epidemic has ended. To this, we also add mean-field transmission. Each generation, the number of new infections is calculated in order to calculate *ε(i)* for each susceptible individual *i*, who is infected from mean field transmission with probability 1 - e ^ε(i)^ each generation.

Recovery from infection is not explicitly modelled in the simulation, but rather is implicitly built into the structure of the model. If an individual *i* is infected in generation *g*, they will infect all of their transmission contacts in generation *g*+1 via the matrix multiplication. They also only contribute to community infection in generation *g*+1. While individual *i* remains ‘infected’ (with value 1), they no longer play any role in the infection dynamics, nor can they be reinfected. Hence, the simulation model assumes that individuals are infectious for one generation, before recovering with immunity.

Results are averages obtained from simulations of 1000 epidemics for 10 different sampled epidemic networks, hence results are averages of 10000 simulations.

### Outcome metrics

We calculate two key metrics for the epidemiological impact of interventions in our household model with extended social contacts, which relate to epidemic risk and adverse health measures.

**Figure 2.**
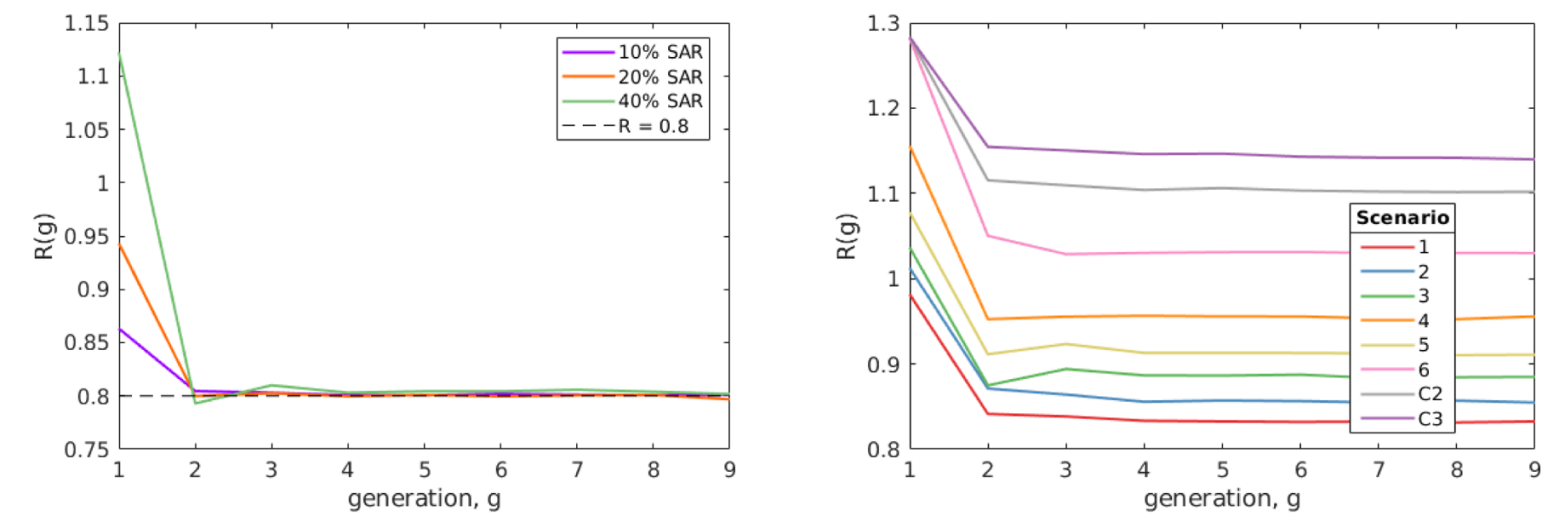
Numerical exploration of *R* by generation. Left shows examples of the method for which ε was fitted to satisfy *R(4)* = 0.8 under our baseline parameters for different values of SAR _HH_. Right shows *R(g)* by generation from each of our scenarios from our baseline assumption. In both plots, *R(g)* decreases over the first few generations, before reaching an equilibrium value that persists over multiple generations.

The net reproduction number ( *R*) is a measure of risk of (increased) transmission that may eventually result in an exponential increase in infections and hence the need for stricter control measures if exceeding the epidemic threshold ( *R>1*). *R* is defined as the number of secondary infections generated by a typical case. In models incorporating household structure, the typical case is effectively an average over the probability that such a case is the first, second, third or later generation case within the household ^44^. Following the principle of Pellis *et al.* ^45, 46^, we determine *R* numerically as the ratio of the number of new infections in the fifth to the fourth model generation, adjusted to account for the partial depletion of susceptibles. Specifically, defining *R(g)* as:

$$R(g)=\frac{\sum_{i}\;I_{g+1}(i)}{\sum_{i}\;I_{g}(i)}\times \frac{N}{\sum_{i}\;S_{g}(i)}$$

we take *R(4)* as *R*. In all simulations this provided sufficient time for the average state of infectious individuals to have stabilised at a value that persists over several generations ( Figure 2). 

Our second metric is the relative mortality (i.e. number of deaths), compared to the baseline model (C1) of isolated households; this provides a measure of adverse health impacts as a result of increased contact rates in the respective scenarios. We use age stratified infection fatality rates (IFR) estimated from repatriation flights early in the COVID-19 pandemic ^42, 47^ to predict the mortality risk in the five generations following model burn in (i.e. from the fourth to ninth model generation - approximately the second month after social bubbles were initiated). Each simulation is initiated with the required number of infectious individuals for 1% of the population to be infected by generation 4, in order for the fatalities following this generation to be meaningfully compared.

### Parameterisation

To parameterise the COVID-19 transmission dynamics in the model we need to define the infection dynamics within a household, within a bubble and from the community. To parameterise the within household transmission we assume that, in line with observations from contact tracing while accounting for some underreporting ^40, 41, 48^, the secondary household attack rate (SAR _HH_) is 20%. This is achieved by tuning the transmission rate ( *τ _H_*) between household members to achieve this average attack rate. We subsequently assume that community transmission is such that, in combination with household transmission, the model generates an overall reproduction number of 0.8, similar to estimates from mid-May 2020 in the UK ^43, 49^. Further, as a base-case, we assume that transmission between households within the same bubble is 50% lower than that within a household, i.e. τ _B_ = ½ * τ _H_. In our base case parameterisation a 3.75-fold increase in community contacts yielded a reproduction number of about 2.5; this is in line with an approximate 70% reduction in contacts during lockdown and a reproduction number of about 2.5 in the early phase of the pandemic with hardly any distancing measures in place ^49^.

We additionally assume that all eligible households would take up the opportunity to expand their contacts and enter into a social bubble with one other household, and that they would adhere to the exclusivity of this bubble. The impact of only partial uptake is explored in our results, and the impact of non-adherence, incorporated by allowing 50% of eligible households to form an additional social bubble, is explored in our sensitivity analyses 5

### Model parameterisation

All analyses were done in MATLAB 2019B ^50^ and R ^51^ v3.6.3 and are available via GitHub ^52^. The R packages reshape2 v1.4.3, tidyverse v1.3.0 , and gridExtra v0.8.1 are required to generate plots. The analysis performed using MATLAB can be replicated using the open MATLAB compatible software GNU Octave, with minimal adjustments, outlined in the GitHub’s ReadMe ^52^.

### Scenarios modelled

We considered a number of contact clustering strategies of how bubbles could be allowed under any relaxation to lockdown measures:

1) Allow households with children younger than 10-years-old (about primary school age or younger) to pair up
2) Allow households with children younger than 20-years-old to pair up
3) Allow single occupancy households to pair up with another single occupancy household
4) Allow adults who live alone or with dependent children only to pair up with another household of any size in a ‘support bubble’
5) A combination of Scenarios 1 and 3
6) Allow all households to pair up with one other household

All these scenarios assume that the pairing will occur at random between permitted households.

We compare the above scenarios against three counterfactuals that do not include social bubbles. These allows us to elucidate the impact of

C1) Perfect adherence to the current household-only contact strategy (other than the background transmission risk from the community)
C2) All individuals increase their number of contacts so that population level force of infection matches that of Scenario 6. Contacts are unclustered and chosen at random across the population but stay the same over time.
C3) All individuals increase their number of contacts so that population level force of infection matches that of Scenario 6. Contacts are unclustered and chosen at random across the population and are re-sampled at each generation.

Counterfactuals 2 and 3, maintain the same level of additional contacts outside the home as social bubbles but change how they are distributed.

C2 is obtained by taking the sampled infectious contacts from Scenario 6, then rewiring these sampled contacts. Doing so keeps the number of secondary infections from any individual constant across both scenarios. As the sampled contact matrices represent transmission networks, edges may be either directed or undirected. Directed and undirected links are rewired separately, so that C2 has the same number of undirected links ( *i* would infect *j* and *j* would infect *i*) as in Scenario 6. C3 is obtained, like in C2, by sampling infectious contacts from Scenario 6 then rewiring these sampled contacts. However, in this situation, all links are treated as directed, and hence the number of undirected links diminishes, reflecting that an individual chooses new bubble contacts each generation. This rewiring reflects that edges are resampled each generation. We also use this method to produce scenario specific counterfactual scenarios, e.g. C2 and C3 for Scenario 1, to assess the effectiveness of bubbling.

### Sensitivity analyses

Other than the previously described base case we performed a number of univariate sensitivity analyses to test the robustness of our findings to the underlying assumptions. Specifically, we assume that the current *R* is 0.7 or 0.9 instead of 0.8 ^43^; that the secondary attack rate in the household is 10% or 40% instead of 20% ^41^; that transmission between individuals in the same bubble (but different households) is 10% or 100% of that within a household instead of 50%; that the risk of a household to get infected with SARS-CoV-2 from the community increases with increasing household size instead of being independent; that transmission across close contacts is density-dependent instread of frequency-dependent; that 50% of bubbles do not adhere to the recommendations but also form bubbles with an additional household rather than perfect adherence; that households including an individual over 70-years-old do not form bubbles; and that the relative susceptibility to infection of children and older adults compared to adults is 79% and 125% while the relative transmissibility is 64% and 290%, respectively ^8, 53^.

We model non-adherence to the strategy by allowing 50% of eligible households to enter into close contact with an additional household. Doing so means that bubbles are no longer mutually exclusive, and that chains of transmission could potentially span many households. Letting *B*_2 _denote the probability matrix of additional bubbles through non-adherence, *A* is now obtained by the sum of *H*, *B*, and *B*_2_.

## Results

### Households

From the 2011 census of England and Wales, the average size of a household was 2.36 persons. Considering households with at least one child under 10-years-old, the average household size increases to 3.89 persons, and 30.4% of the population live in such households. For households with at least one child under 20-years-old, the average household size is 3.73 persons, and 49.5% live in such households. In total, 37% of households are occupied by someone over the age of 60 years, and 50% of single occupancy households were occupied by such older adults. Single occupancy households comprise 30.2% of households. There is limited multi-generational mixing, with only 3.6% of households having both a child aged under 10 years and an adult aged over 60 years.

### Impact of social bubble strategies on epidemic risk

Assuming an initial reproduction number of 0.8, perfect adherence to the recommended social bubble strategy and that all eligible households indeed pair up, we find that strategies that exclusively target single-person households (scenario 3) or households with young children (scenario 1) do not increase transmission substantially ( *R* of 0.83 and 0.89 respectively in the base case scenario); their combination (scenario 5) is also predicted to only marginally increase transmission in the community ( *R* of 0.91) ( Figure 2). For these two targeted strategies, even under conservative assumptions (SAR _HH_ = 40%, τ _H_ = τ _B_), the increase in transmission is unlikely to lead to substantial spread of COVID-19 ( *R* of 0.95 and 0.91 for scenario 1 and 3, respectively).

**Figure 3.**
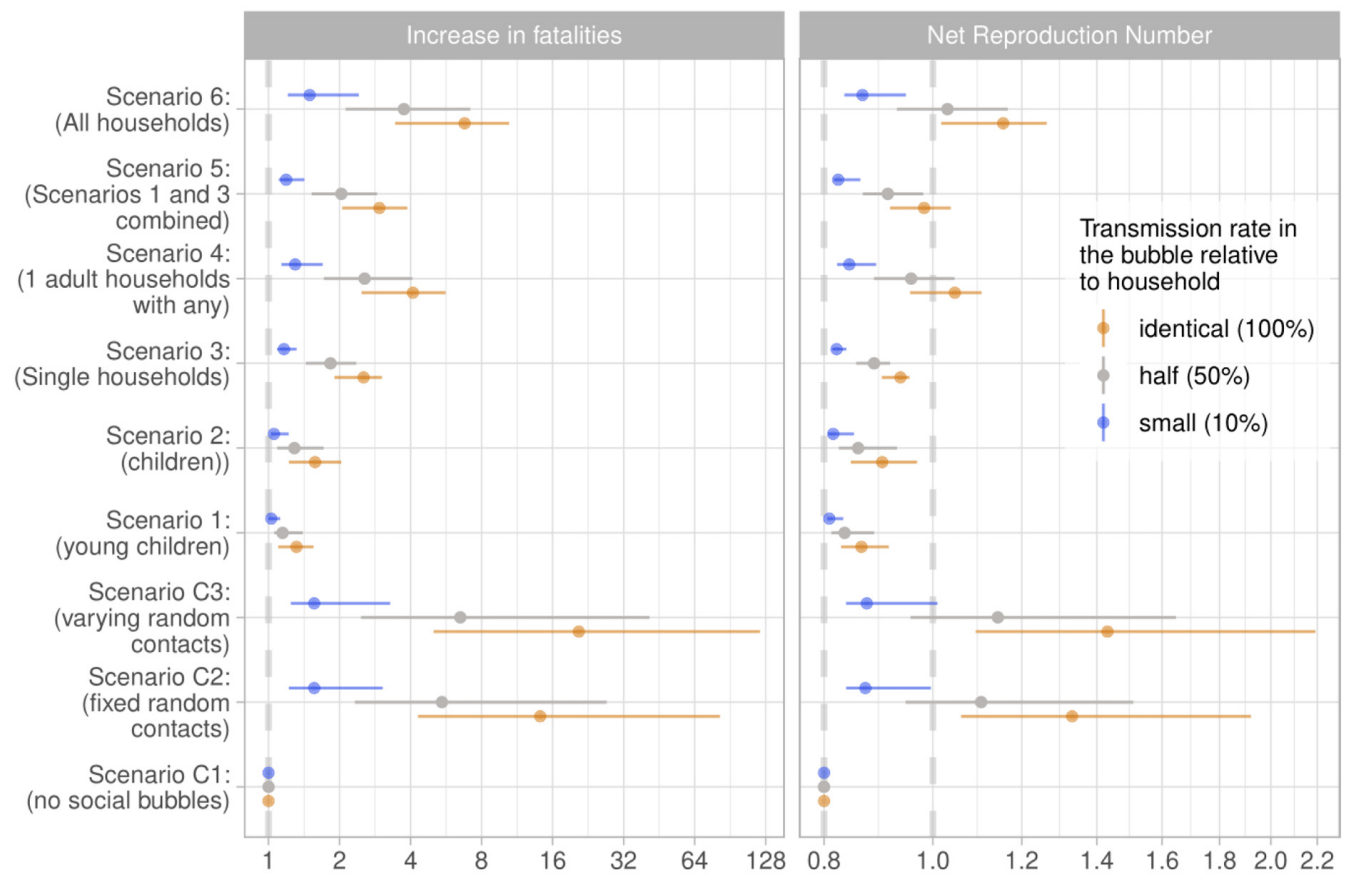
Estimated reproduction number and increase in fatalities for the considered scenarios under the assumption that all eligible households pair up and thereby form exclusive social bubbles and that transmission rates within a social bubble are the same as within the household. Central estimates are assuming SAR _HH_=20% and the upper and lower limits represent the respective 10% and 40% assumption.

However, allowing all households to form bubbles (scenario 6) is estimated to increase the reproduction number to 1.02, and hence beyond the critical threshold value of 1 for the base case scenario ( Figure 3).

**Figure 4.**
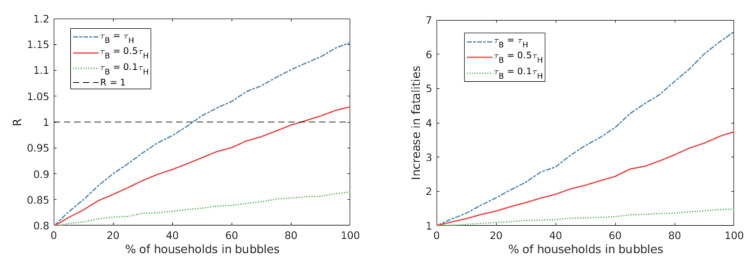
The impact of uptake on *R* and fatality. Here we consider the impact varying levels of uptake has on the reproduction number, *R*, and relative mortality. We consider this for our baseline parameters, at varying levels of transmission across bubble contacts (τ _B_ = τ _H_ in blue, τ _B_= 0.5 τ _H_ in red, τ _B_ = 0.1 τ _H_ in green). We observe that *R* scales sublinearly with uptake, with the gradient of increase dependent on transmission rate across bubble contacts.

Generally, the fewer households that were deemed eligible for expanding their social bubble under a specific strategy, the smaller the average household size of those involved. and the smaller the risk of transmission within the bubble, the smaller the increase in transmission as a result. The impact of social bubbles also depends on uptake - we find that *R* increases sublinearly with uptake ( Figure 4).

**Figure 5.**
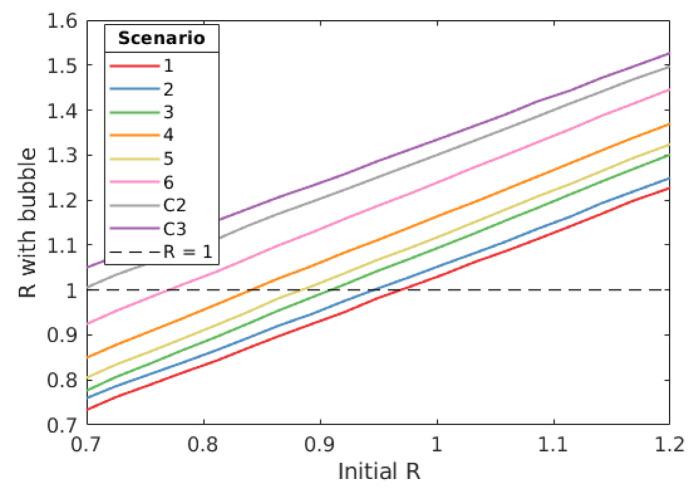
The relationship between initial *R* and *R* under different bubble scenarios. Here we consider the impact different bubble strategies have on the reproduction number, *R*. We consider this for our baseline parameters. For scenarios 3 to 6, we find that *R* with bubbles increases linearly with initial *R* with a gradient above 1, meaning that as initial *R* increases, the greater the increase bubble strategies have on *R*.

The impact bubbles have on epidemic risk depends upon the levels of transmission within the population prior to introducing a bubble strategy. We find that the impact of bubble strategies on transmission scales linearly with the prior *R* value *, but* for some strategies with a gradient > 1, meaning that the higher the level of community transmission within the population the larger the increase in *R* from allowing social bubbles ( Figure 5).

### The impact of social bubble strategies on mortality risk

**Figure 6.**
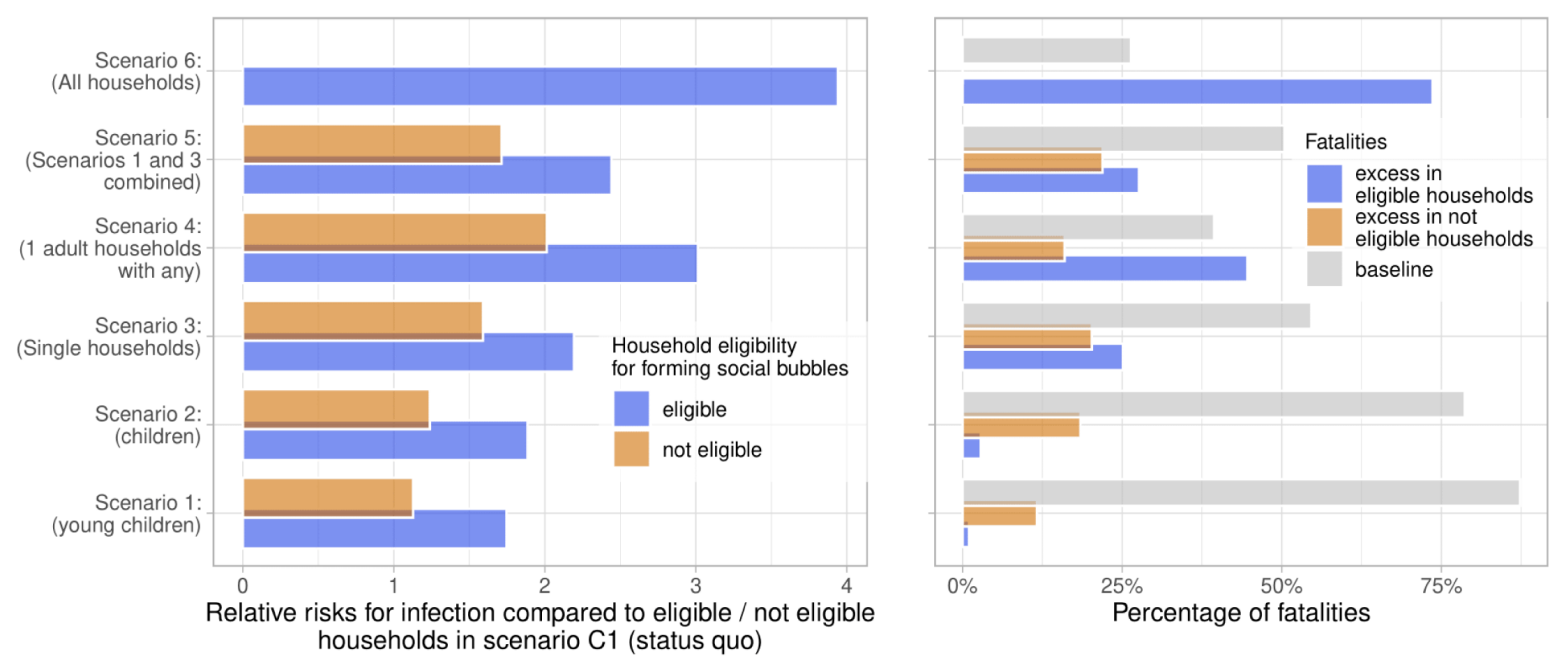
Relative risk of infection and fatality. Left panel: the relative risks for infection in the considered scenarios if compared to the status quo with no social bubbles (Scenario C1), stratified into the risks in households eligible and not eligible for forming social bubbles. Right panel: the population attributable fraction of fatalities in the considered scenarios. The overall mortality risk is stratified into the baseline risk, and the excess risk from forming social bubbles in both eligible and non eligible households.

The average age in the households eligible to form social bubbles in scenarios 1 to 6 was 21.8, 25.6, 58.1, 40.2, 32.2, and 39.4 years, hence the average infection fatality risk in an average household member implementing such a strategy was 0.09%, 0.14%, 2.36%, 1.05%, 0.74%, and 0.93%. In all scenarios, the increased number of contacts lead to both excess infections and fatalities. Excess risk for infection compared with no social bubbles (Scenario C1) was seen in households implementing the social bubbles as well as those households who were not eligible, although, as expected, the relative risk for infection was higher in eligible households ( Figure 6). The resulting excess mortality risk depended highly on the estimated epidemic risk but also on the average age of the affected households.

For example, while social bubbles among households with young children (Scenario 1) saw the similar increases in infections to increases in deaths (with a risk ratio of 1.13 and 1.14 for infections and deaths respectively), social bubbles targeting single occupancy households saw a larger increase in deaths than infections (with a risk ratio of 1.26 and 1.83 for infections and deaths respectively) due to the older targeted demographic. In scenarios targeting families, the mortality risk was largely attributed to households not eligible to form social bubbles ( Figure 6).

### Effectiveness of social bubbles

The forming of social bubbles was effective at reducing the infection and thereby the mortality risk compared to strategies that increased contacts in a less clustered way: under base case assumptions all households forming social bubbles (Scenario 6) reduced the mortality risk by 30.9% and 42.4% compared to adding the same amount of contacts randomly (Scenario C2) and time varying (Scenario C3).

**Figure 7.**
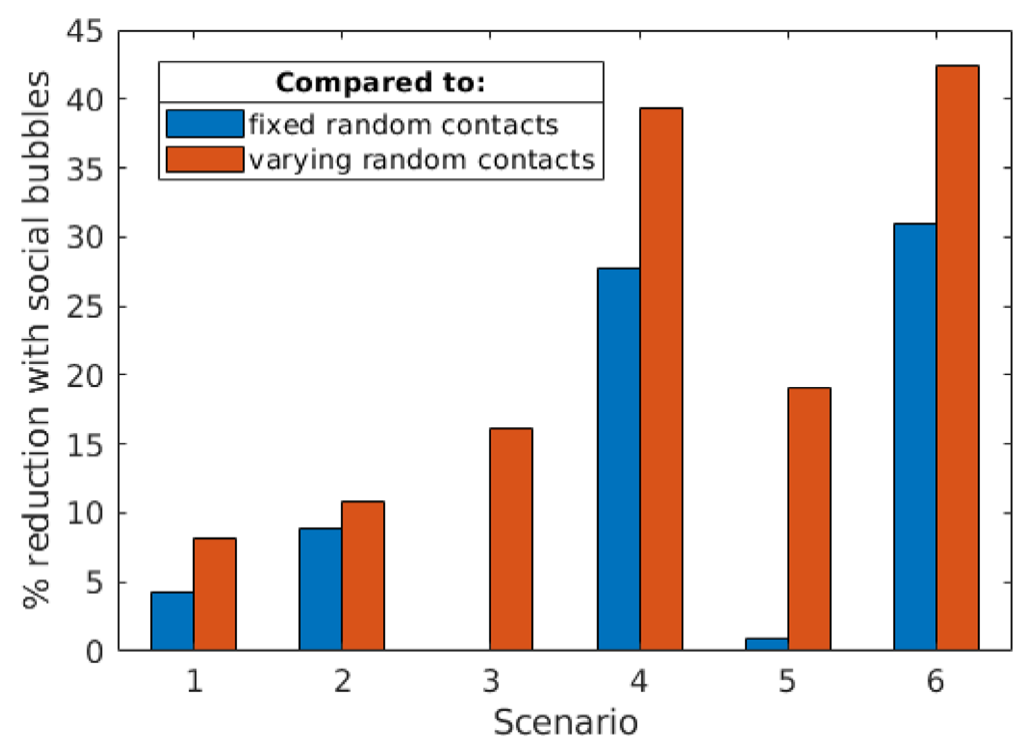
Scenario specific effectiveness of social bubbles. Here we compared the effectiveness of social bubbles in reducing mortality risk, when compared to other ways of increasing social contacts - where individuals from eligible households either make fixed random contacts (blue) or varying random contacts every generation (orange). In each comparison, individuals make the same number of infectious contacts, so the reduction in fatalities can be attributed to the clustering implied by social bubbles.

In general, the added benefit of social bubbles increases with the higher proportion of eligible households, alongside targeting riskier populations. For example, social bubbles for households with young children (Scenario 1) reduced mortality risk by 4.2% and 8.1% compared to those households increasing contacts randomly and time-varying. In contrast, allowing households with one adult to form a support bubble with another household (Scenario 4) results in 51% of the households entering into a bubble, and leads to a 27.7% and 39.3% reduced mortality risk compared to those households increasing their contacts randomly and time-varying respectively ( Figure 7).

### Sensitivity analyses

**Figure 8.**
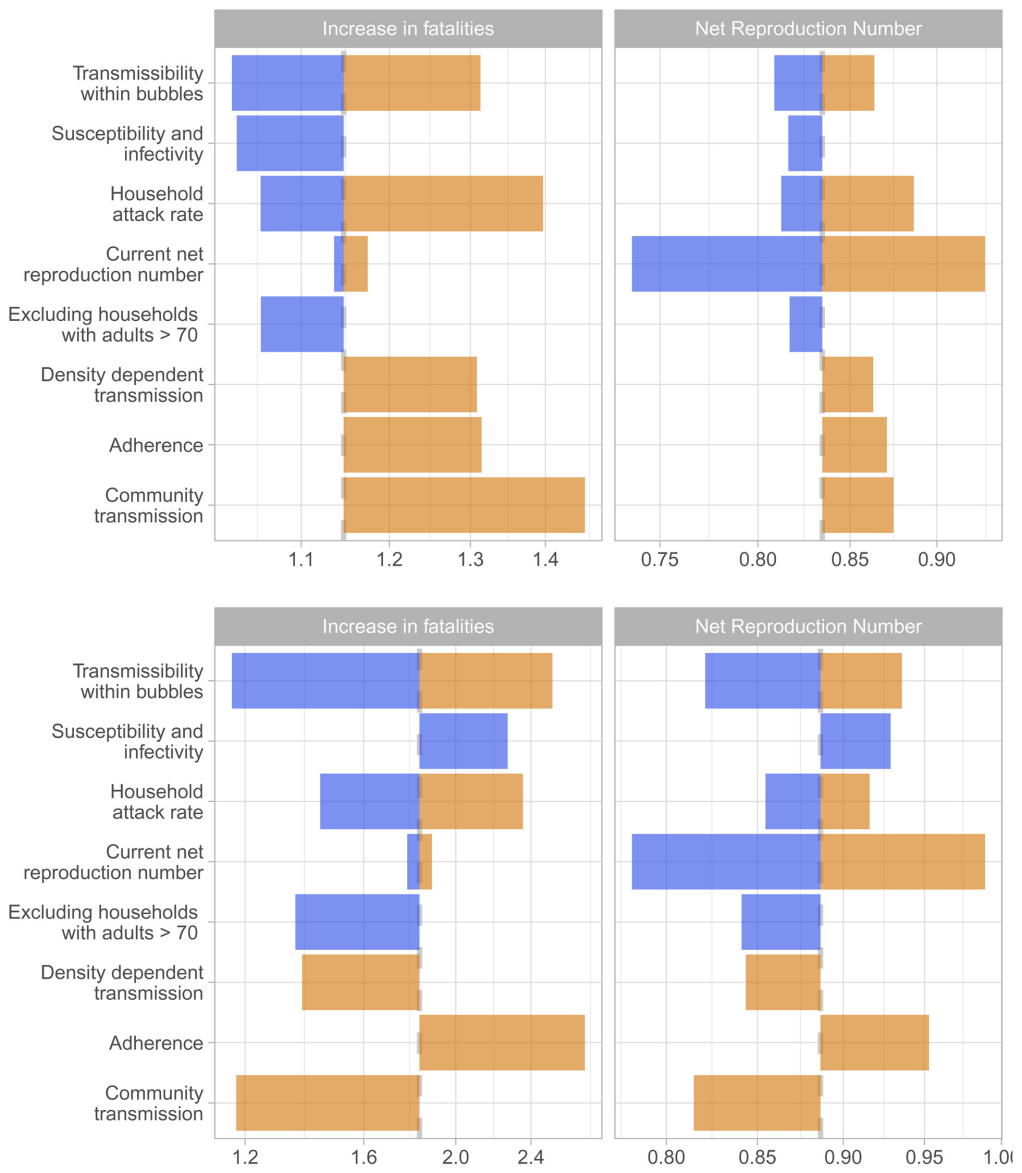
Sensitivity analyses. The tornado diagram shows a univariate sensitivity analysis on the expected increase in fatalities and the net reproduction number for scenario 1 (above) i.e. allowing households with young children to pair up, and for scenario 3 (below), i.e. allowing single occupancy households to pair up. The color coding is based on factors determining higher risk (orange) and lower risk (blue) for Scenario 1. The base case estimate is indicated through the dashed grey vertical line. The sensitivity scenarios are (from top to bottom): transmission across individuals of households sharing a bubble is 90% or 0% lower than that within a household instead of 50%; the relative susceptibility to infection of children and older adults compared to adults is 79% and 125% while the relative transmissibility is 64% and 290%; the secondary attack rate in the household is 10% or 40% instead of 20%; *R _e_* is 0.7 or 0.9 instead of 0.8; that households including an adult over 70-years-old are excluded from forming bubbles; density-dependent transmission across close contacts instead of frequency-dependent transmission; that 50% of bubbles do not adhere to the recommendations but pair up with an additional household; and that the risk of a household to get infected from the community is proportional to the household size instead of being the same across households.

We tested the robustness of our findings to a number of alternative assumptions governing the spread of SARS-CoV-2 and the implementation of the social bubble strategy. Within the tested parameter space, the alternative assumptions did not qualitatively change our findings. The two main factors that increased or decreased the epidemic risk were an initial value of *R* closer to 1 when implementing the strategy and a much higher than typically observed secondary household attack rate. However, for Scenarios 1 and 3, in neither of the univariately tested parameterisations did *R* exceed 1 ( Figure 8). The assumptions on age stratified susceptibility and transmissibility were conservative for strategies focussed on households with children and were optimistic for single-person households; and vice versa for the assumption that the risk for community transmission was independent of household size. The epidemic risk from social bubbles is further reduced if within bubble transmission is reduced to 10% of that within household transmission. The alternative assumptions surrounding close-contact transmission and community transmission changed the ordering of risk of social bubble strategies. Under our baseline assumption, Scenario 1, targeting families with young children, resulted in the lowest increase in R; under these alternative assumptions, Scenario 3, targeting single-occupancy households, resulted in the lowest increase in R. The corresponding tornado diagrams for all other scenarios are included as extended data ^52^.

The effectiveness of social bubbles also varied according to the underlying parametric assumptions. Assuming our alternative assumptions around susceptibility and infectivity, the effectiveness of social bubbles was as large as a 46.1% and a 58.5% reduction in mortality risk compared to adding the same amount of contacts randomly (Scenario C2) and time varying (Scenario C3). Under our most conservative assumptions, the reductions in mortality risk compared to C2 and C3 were 87.2 and 91.3%.

Alongside the parameter sensitivity scenarios considered, we also considered each scenario where older adults were shielded and excluded from being allowed to form a bubble as a sensitivity analysis. This only has a small impact on the effect of social bubbles for families with children (Scenarios 1 and 2), because of the small amount of multi-generational mixing between households in the UK, but does reduce *R* for social bubbles for single occupancy households or all households (Scenarios 3–6). While shielding older individuals does decrease overall mortality risk, such a strategy still impacts older individuals; bubbling strategies increase overall cases, which in turn increases risk to older individuals through community transmission.

 Another potential strategy would be to allow all households with two or fewer adults to form a bubble with households of any size - an extension to the current situation in England. However, as 87.7% of households have two or fewer adults, such a policy would result in 98% of households forming bubbles, and hence such a policy would have largely the same impact as allowing all households to form bubbles.

## Discussion

We found that contact clustering, or the forming of social bubbles that join two households, can allow increased social contacts beyond the households while limiting additional risk of transmission. In the base case social bubbles reduced the mortality risk by 42% compared to a scenario that increased contacts by the same amount but without clustering thereof, and risk reduction under some alternative parameterisations was even higher. Allowing all households to form social bubbles may increase *R* above its epidemic threshold and hence lead to an increase in cases. A strategy that sees only those at potentially the highest need for an extension of their contacts beyond the household (families with young children and single-person households) should lead to a limited increase in epidemic risk (less than 11% individually and less than 15% in combination), which remained below the epidemic threshold in most scenarios considered. The epidemic risk can be further reduced if the transmission risk within the bubble is minimised. As the number of contacts and *R* increase with a social bubble strategy, so does the risk of adverse health outcomes. We find that adverse health outcomes are largely proportional to the epidemic risk, but will disproportionately affect households with older adults independently of their clustering behaviour.

Stringent physical distancing policies in many countries have reduced *R* from about 2.5 to just under 1 ^11, 49, 54^. This provides the opportunity to risk a small amount of additional contacts without necessarily experiencing an increase in COVID-19 cases, if crossing the epidemic threshold can be avoided. Here, we investigate the effectiveness of social bubbles as a potential option to ease the social impact of the lockdown without increasing transmission risks. However, while we consider the impact of social bubbles in isolation, such a policy would only be one part of a multi-variable exit strategy ^28^. Hence, our comparisons of alternative bubble strategies against the epidemic threshold should be interpreted cautiously and in consideration of the other changes to behaviour, such as re-opening of non-essential retail and travel. It is likely that these other activities will combine in a non-linear manner so that bubbling in a context of children returning to school might affect results.

Countries including Germany and New Zealand have implemented strategies similar to those considered here. In Bavaria, Germany, in early May and before the reopening of schools and nurseries, up to three households could form exclusive groups to share childcare amongst them ^55^. Even during their highest national alert level, level 4 “Lockdown”, New Zealand permitted people living alone to pair up with a “lockdown buddy” and key workers to identify “childcare buddies”. New Zealand moved to level 3 in their COVID-19 alert system, “Restrict”, on 27 April 2020, which included the advice to residents to stay within their household bubbles but permitted expansion of such to reconnect with close family, bring in caregivers or support isolated people ^56^. A subsequent survey found that among respondents the highest increase in the quality of life by far would not be brought by re-opening of schools, shops, churches or fitness centres, but by allowing households to re-connect ^57^. It also found that in going to alert level 3 only 50% of households took up the opportunity to expand their social bubbles and that there was high awareness of the importance of the exclusivity of the bubbles, with only 7.5% of bubbles reporting to have had contacts outside their bubble.

We identify three key risks to the success of social bubbles that may increase their epidemiological risk: potential lack of adherence, a higher than observed secondary household attack rate and being too close to the epidemic threshold. If the risk perception of the population changes as a result of allowing parts of the population to form social clusters, a lack of adherence to the exclusivity of the bubbles could lead to rebuilding of contact networks that in turn lead to the epidemic threshold being crossed. We find that some degree of non-adherence would not necessarily hinder the success of the strategy, but communication of the strategy is likely to be key. For example, in New Zealand, the social bubbles were not framed as a relaxation of social distancing rules but rather as a source of support for those who are at a higher risk of social isolation or with needs for care, including childcare ^57^. We find that if the secondary household attack rate is substantially higher than assumed in our base case the epidemic risk is elevated close to the epidemic threshold. There remains some uncertainty surrounding the household attack rate of COVID-19, with high household attack rates observed in some instances. However, our base case assumptions are in line with an increasingly consistent picture emerging in the contemporary academic literature ^41^. Also, superspreading events have been raised as a potentially important source for sustained transmission of SARS-CoV-2, which would further imply a rather low secondary household attack rate in most instances ^58, 59^. However, household attack rates may vary between different types of household, and may be larger for some households with unusual network structures ^60^, such as large student households. Similarly, if the R is very close to its epidemic threshold, an increase in contacts, even if clustered, could result in an increase in cases. Hence, careful monitoring of such is needed to assess the feasibility of expanding social bubbles.

An expansion of contacts into social bubbles will naturally lead to some increase in transmission in comparison to perfect adherence to the recommendation to restrict to all but essential contacts outside the household. However, such adherence may decline as a result of extended periods of time in lockdown and lead to an expansion of contacts that are unclustered, potentially leading to long chains of transmission. To illustrate such a scenario, we include alternative comparisons for the strategy that allows all households to form social bubbles (Scenario 6). We considered strategies that would have the same overall increase in transmission as in that scenario but where either the contacts or not cluster but stay fixed over time (Scenario C2) or where contacts are not clustered and vary over time (Scenario C3). We show that the clustering reduces the epidemic and reduces the number of infections and subsequent fatalities by 30.9% and 42.4% in the base case and even more in some of the parametric sensitivity analysis. Hence social bubbles, if given as a guidance to households who are struggling to cope with the lockdown, may give these households a safer alternative and thereby help to reduce the epidemic and mortality risk. This may particularly be the case for households with single parents or parents who cannot easily work from home; in such circumstances allowing social bubbles may help increase equity in the impact of the lockdown.

As in any epidemiological modelling study, we must make some assumptions surrounding transmission. Firstly, we assumed that transmission across close contacts was frequency dependent, informed by previous studies that indicate the probability of infection across two specific members of the same household decreases with household size for COVID-19 (and other communicable diseases like influenza). However, there remains uncertainty surrounding the nature of close-contact transmission for COVID-19, and the assumption of frequency dependence may not accurately capture transmission across all settings. Secondly, we assumed that the risk of a household acquiring infection from the community is independent of its number of occupants. While this assumption may be appropriate for some households (e.g. families where one adult leaves the household to do shopping), it may not hold true in other contexts (e.g. student households comprising of largely independent individuals). Because of this we tested the sensitivity of our results to these assumptions by alternatively considering close contact transmission as density dependent, and allowing the risk of household infection to increase with household size. These alternative assumptions do not qualitatively change our findings, but do change the ordering of the risk of social bubble strategies. In particular, under either of these alternative assumptions, social bubble strategies targeting single occupancy households result in a lower increase in R than strategies targeting families with children. Reports on the antibody prevalence in England found household size associated with antibody prevalence, suggesting that household size may play a role in the probability of acquiring infection ^61^; a more detailed understanding of the nature of close contact and community transmission may help inform more precise evaluations of the effectiveness of social bubbles. We do not consider the risk of community transmission depending on bubble size. However, if bubbles were to act as cohesive units, and as a consequence reduce their interaction with the community, this may further increase the effectiveness of social bubble strategies.

Our analyses have a number of limitations. Firstly, we only assessed the risk of extending social bubbles but not the benefits. As of June 2020 in England, social contact beyond the immediate household is restricted to virtual contact or contact in open spaces with up to five individuals while keeping 2 metres apart. In other words, one can have a conversation. While conversations are a large part of the social contacts of adults they have little role in the social interactions of young children. Hence the benefit of extending bubbles for children is likely disproportionately higher. Furthermore, clustering contacts into social bubbles is likely to ease contact tracing, which is an integral part of both containment and lockdown exit strategies. We considered social bubbles against the background of a lockdown, particularly where schools are closed. As lockdown measures are eased and schools are gradually re-opened forming social bubbles that largely overlap with societal contacts (e.g. forming social clusters with families that have children going to the same class) is likely further reducing the additional epidemic risk from social bubbles. By the same token, as lockdown eases, social bubbles aligned with other types of contacts, such workplace contacts or sport team contacts, would likely improve the efficacy of any bubbling strategy ^27^. We also did not include the possibility to form bigger social bubbles that would cluster together three or more households. While this has been implemented in other countries, the complexity of creating an exclusive cluster of three or more households could lead to a loss of adherence. We did not consider further heterogeneity within society that may affect both risk of transmission and adverse health outcomes. For example, about 20% of the working population is classified as key workers and will have an increased risk for infection from the community, while adverse health outcomes have disproportionately affected individuals of low socioeconomic status. Further, we did not consider age-homogeneous mixing when pairing up households into bubbles, which may lead to a further layer of clustering and thereby reduce the mortality risks associated with the bubble strategy.

## Conclusions

Our analyses highlight the continued need for social distancing despite a social bubble strategy being an effective way to expand contacts while limiting the risk of a resurgence of cases. Recommending social bubbles only for those who particularly struggle with a lockdown, while minimising opportunities for spread through prioritising outdoor settings for gatherings and adhering to distancing recommendations as much as possible, may strike an effective balance between minimising the impact on mental health and minimising the risk of a resurgence of cases. With the increased number of local lockdowns and the risk of a second wave in the Winter of 2020, social bubbles may again become a vital tool to provide social interactions to those that need it most whilst keeping *R* below one.

## Data availability

### Source data

The synthetic population for the study was constructed by from the 2011 (27 March) census, from the table ‘ *SN 8637 - 2011 Census Ad Hoc Household Composition (Age Groups) Safeguarded Tables (Lower Layer Super Output Area): England and Wales.* This table is safeguarded, and can only be accessed by approved researchers. However, we have included our synthetic population in our repository as a Matlab workspace, ‘FullCensusHouseholdWorkspace.mat’, which can be used to regenerate results and to derive population level statistics. Alternatively, Office for National Statistics data for the age distribution of households in ten year age bands containing six individuals or fewer are available as dataset CT1088, ( https://www.ons.gov.uk/search?:uri=search&:uri=search&q=CT1088*). Households of size six or less account for 98.2% of the households in England and Wales, and contain 97.8% of their combined population, so the loss accuracy induced by this cutoff is likely to be minimal.

### Underlying data

The simulation model and analyses from this study are available via GitHub https://github.com/tsleng93/SocialBubble/tree/v1.1

Archived code at time of publication: http://doi.org/10.5281/zenodo.4605475 ^52^

To generate the underlying data for the plots in Figures 3, 8, and the extended data, users should run the code 'MainCode.m'. After running this, users should then run 'DataMaker.m' to produce the underlying .csv files.

To generate the underlying data ^52^ for the plots in Figure 6, users should run the code 'figure6Code.m', which runs the code and produces the underlying .csv files.

The R code 'plots.r' can be run as it is in order to generate the exact plots of Figures 1, 3, 6, 8 and the extended data ^53^. To generate analogous plots from regenerated data, the .csv files should be replaced with those generated from 'MainCode.m' and 'figure7Code.m'

To generate the data and plots for Figure 2, Figure 4, Figure 5, and Figure 6 users should run the code 'figure2Code.m', 'figure4Code.m', etc.

### Extended data

Zenodo: tsleng93/SocialBubble: SocialBubble. http://doi.org/10.5281/zenodo.4013702 ^52^

This project contains the following extended data:

- main_tornado_Scenario_2.pdf (univariate sensitivity analysis for Scenario 2)
- main_tornado_Scenario_4.pdf (univariate sensitivity analysis for Scenario 4)
- main_tornado_Scenario_5.pdf (univariate sensitivity analysis for Scenario 5)
- main_tornado_Scenario_6.pdf (univariate sensitivity analysis for Scenario 6)
- main_tornado_Scenario_C2.pdf (univariate sensitivity analysis for Scenario C2)
- main_tornado_Scenario_C3.pdf (univariate sensitivity analysis for Scenario C3)

Data are available under the terms of the Creative Commons Zero "No rights reserved" data waiver (CC0 1.0 Public domain dedication).
