# Supplementary material for: The effectiveness of social bubbles as part of a Covid-19 lockdown exit strategy, a modelling study
Source: Wellcome Open Res. 2021 Mar 29;5:213. Originally published 2020 Sep 10. [Version 2] doi: 10.12688/wellcomeopenres.16164.2 (PMC7871360; doi:10.12688/wellcomeopenres.16164.2)
Supplement: Peer review history

# Peer review: Reviewer response for version 1

**DOI:** 10.21956/wellcomeopenres.17747.r42171

**Published:** 2021-02-08

**Authors:** Martin C. J. Bootsma

**Affiliations:** 1 Julius Center for Health Sciences and Primary Care, University Medical Center Utrecht, Utrecht University, Utrecht, The Netherlands; 2 Department of Mathematics, Faculty of Sciences, Utrecht University, Utrecht, The Netherlands

The authors have developed a model with which they can investigate what the effect is if two household are allowed to form a bubble. The authors analyse this for different groups of households which are allowed to form bubbles and compare this to scenarios without bubbles and if the additional contacts do not occur in bubbles but randomly.

 I think the models are suitable for the research question. As the code is provided by the researchers. the results can be replicated and other research questions could be addressed by the model as well.

 I have only minor points, mainly things that could be clarified to make it easier for readers to understand the methodology.

 On page 3 “connections are captured by a matrix, A, which defines the probability

 that infection can pass between any two individuals in the population. A”. I think that the authors should specify what the nature of this probability is and that the model uses generations and that the probabilities refer to probabilities of transmission during the infectious period (and not a probability per day, for example).

 I was surprised by the assumption “transmission within households and across households who share a bubble is frequency dependent.” And wondered whether it would be reasonably to perform a sensitivity analysis to this (assuming a density dependent transmission rate), as the risk of transmission within a household and in bubbles in some cases, say sitting around one table, may not be frequency dependent.

 In table 1: regarding ρH(i,j) specify what the unit of time is for the rate (duration infectious period).

 The authors may consider to use a different symbol than C which already in use as  the age-dependent sensitivity.

 In Table 2, check the use of \tau and T

  The authors claim to have performed a sensitivity analysis for the case “that the risk of a household to get infected with SARS-CoV-2 from the community increases with increasing household size instead of being independent”. I did not see the results in the manuscript or the online-material. Did I miss this?

Is the work clearly and accurately presented and does it cite the current literature?

Yes

If applicable, is the statistical analysis and its interpretation appropriate?

Yes

Are all the source data underlying the results available to ensure full reproducibility?

Yes

Is the study design appropriate and is the work technically sound?

Yes

Are the conclusions drawn adequately supported by the results?

Yes

Are sufficient details of methods and analysis provided to allow replication by others?

Yes

Reviewer Expertise:

Mathematical modeler of infectious disease dynamics

I confirm that I have read this submission and believe that I have an appropriate level of expertise to confirm that it is of an acceptable scientific standard.

# Response

**Published:** 2021-03-15

**Authors:** Trystan Leng

**Affiliations:** University of Warwick, UK

We thank both reviewers for their considered and detailed reviews of our manuscript. We

 respond to each of the points raised by reviewers below, and have also revised the main body

 of the manuscript where appropriate.

 Reviewer 2:

- Comment: ​ On page 3 “connections are captured by a matrix, A, which defines the probability that infection can pass between any two individuals in the population. A”. I think that the authors should specify what the nature of this probability is and that the model uses generations and that the probabilities refer to probabilities of transmission during the infectious period (and not a probability per day, for example).
  Response: ​ We agree that this should be clarified. We have amended the above sentence to (added text in bold:)
  “This is achieved through stochastic simulation of infections spreading through an interconnected population ​ **by generation​ **; connections are captured by a matrix, A, which defines the probability that infection can pass between any two individuals in the population ​over **an individual’s entire infectious period​ .**”
- Comment: ​ I was surprised by the assumption “transmission within households and across households who share a bubble is frequency dependent.” And wondered whether it would be reasonably to perform a sensitivity analysis to this (assuming a density dependent transmission rate), as the risk of transmission within a household and in bubbles in some cases, say sitting around one table, may not be frequency dependent.
- Response: ​Our decision to assume frequency-dependent transmission for close contacts was based upon studies indicating that the probability of infection across two specific members of the same household decreases with household size for COVID-19 (and other communicable diseases like influenza). However, we acknowledge that for COVID-19 the nature of within-household transmission remains relatively uncertain, and that whether transmission within households is frequency or density dependent may depend on the specific context of the household. We have performed this sensitivity analysis - finding that the assumption of density-dependence does not qualitatively change our results (although does change the ordering of scenarios - with density dependent transmission, scenario 3 (single-occupancy household bubbles) now results
  in the smallest increase in R. We have adjusted the text in the methods, the results (Figure 8), and the discussion to indicate we have considered density-dependent transmission. The added text in the discussion is included in bold below:
**“As in any epidemiological modelling study, we must make some assumptions surrounding transmission. Firstly, we assumed that transmission across close contacts was frequency dependent, informed by previous studies that indicate the probability of infection across two specific members of the same household decreases with household size for COVID-19 (and other communicable diseases like influenza). However, there remains uncertainty surrounding the nature of close-contact transmission for COVID-19, and the assumption of frequency dependence may not accurately capture transmission across all settings.**
** Secondly, we assumed that the risk of a household acquiring infection from the community is independent of its number of occupants. While this assumption may be appropriate for some households (e.g. families where one adult leaves the household to do shopping), it may not hold true in other contexts (e.g. student households comprising of largely independent individuals).. Because of this we tested the sensitivity of our results to these assumptions by alternatively considering close contact transmission as density dependent, and allowing the**
** risk of household infection to increase with household size. These alternative assumptions do not qualitatively change our findings, but do change the ordering of the risk of social bubble strategies. In particular, under either of these alternative assumptions, social bubble strategies targeting single occupancy households result in a lower increase in R than strategies targeting families with children. Reports on the antibody prevalence in England found household size associated with antibody prevalence, suggesting that household size may play a role in the probability of acquiring infection; a more detailed understanding of the**
** nature of close contact and community transmission may help inform more precise evaluations of the effectiveness of social bubbles.”**
- Comment: ​ The authors may consider to use a different symbol than C which already in use as the age dependent sensitivity.
  Response: Thanks for this suggestion. We have changed C in row 6 of table 1 to k.
- Comment: ​ In Table 2, check the use of \tau and T
  Response: ​ We apologise for this typesetting error. Capital T should refer to the transmissibility of an individual i, whereas transmission rates should be τ’s - this has been amended and notation has now been used consistently throughout the manuscript.
- Comment: ​ The authors claim to have performed a sensitivity analysis for the case “that the risk of a household to get infected with SARS-CoV-2 from the community increases with increasing household size instead of being independent
  Response: ​ This appears as the ‘Community transmission’ row of the tornado diagrams of the sensitivity analyses. We now discuss the sensitivity analyses that change the ordering of scenarios (the alternative assumptions surrounding community transmission
  and density dependent transmission) in Results subsection ‘sensitivity analyses' (added text in bold:)
**“The alternative assumptions surrounding close-contact transmission and community transmission changed the ordering of risk of social bubble strategies.**
** Under our baseline assumption, Scenario 1, targeting families with young children, resulted in the lowest increase in R; under these alternative assumptions, Scenario 3, targeting single-occupancy households, resulted in the**
** lowest increase in R.”**

# Peer review: Reviewer response for version 1

**DOI:** 10.21956/wellcomeopenres.17747.r40386

**Published:** 2020-10-14

**Authors:** Jessica Enright

**Affiliations:** 1 School of Computing Science, University of Glasgow, Glasgow, UK

This paper describes a careful consideration of the potential for between-household social bubbling to contribute to Covid-19 control during the current (2020) pandemic. The main narrative comparison is between a lockdown scenario where most contact is within a household as compared to several different bubbling arrangements, but a variety of scenarios are considered beyond bubbling to assess bubbling efficacy. The authors conclude that within their modelling framework a variety of bubbling arrangements would feasibly keep the reproductive number below 1 (thus preventing exponential growth of infections). This is a valuable piece of work that I hope has contributed to the evidence for policymaking during emergence from lockdown after the first wave of the pandemic in the UK. To my understanding, a scenario closest to Scenario 4 is what was actually implemented within most of the UK.  

 The authors have done a good job of incorporating the available knowledge about the epidemiology of Covid-19 at the time they wrote this paper. Given the pace of developments it would have been impossible to incorporate all emerging information, and certainly more knowledge has emerged since the publication of this work that interacts with the work and conclusions here - I don’t think it would be fair to request the authors try to incorporate these subsequent developments.  

 To my best understanding, the model is based on a generation matrix combining household and bubble contacts that is subsampled using transmission probabilities to give contacts that would result in infection. In addition there is mean-field infection across the population. Parameters are from a combination of published sources, adjustments to produce observed R values, and values chosen for sensitivity analysis.   

 The counterfactual approach to comparison in which the number of infectious contacts are maintained but are rewired outside of bubbles or households in C2 and C3 is useful, and it is unsurprising that these counterfactuals results in more overall infection. I was initially slightly puzzled by the use of directed edges here (given that a single contact could transmit in either direction), but on reflection it makes sense in the context of the subsampling of the matrix to give essentially a transmission network rather than a contact network. The authors may wish to include a sentence or two clarifying this choice.  

 I would also have been interested in seeing an analysis of bubbling value in a setting with a steadily increasing mean-field risk of transmission, as an attempt to model a general opening-up and increase in activity. However, I realise this is beyond the scope of this work, and I note that the authors discuss this in the Discussion, noting the likelihood of a ‘multi-variable exit strategy’ in which bubbles might combine with other restriction easements.  

 I wanted to highlight an assumption that I think is important for this model, and that may not apply in all situations: the idea that “We therefore assume that the risk of a household acquiring infection from the community is independent of its number of occupants”. While I think this is well-supported by citations and is appropriate within the setting considered here, I just wanted to highlight that this may not hold true in larger households composed of multiple unrelated adults - e.g. in student households.  In the REACT-2 reports of antibody prevalence (https://www.medrxiv.org/content/10.1101/2020.08.12.20173690v2 ^1^) after several months of the pandemic in England, individuals in larger households were more likely to have antibodies - of course this could also be potentially explained by a fixed within-household SAR regardless of household size, and the many risk-factors captured within REACT-2 correlated with living in a large household. The authors include a violation of this assumption in the sensitivity analyses, and if I have understood correctly it doesn’t qualitatively change the findings. I also found it interesting that the authors chose to have the mean-field transmission depend on an individual’s household size, but not on bubble size - would we expect bubble interactions to decrease interactions in the community that would contribute to the general spread modelled as mean-field here? Am I correct in thinking that moderating the mean-field transmission including bubble size would have increased the efficacy of bubbles?

 The authors note that the epidemic risk was sensitive to a much higher than observed secondary attack rate within a household - I wanted to note that this parameter is still somewhat uncertain, though their estimate is very reasonably based in observation. Again (and this does not detract from the application of this work to a general population), this may differ in very unusual e.g. large young-adult student households due to differing network effects within the household (for example, as described in https://rss.onlinelibrary.wiley.com/doi/10.1111/rssc.12011 ^2^).

 There are many possible extensions to this work that could take it beyond the emerging-from-lockdown scenario - the authors mention that in the case of schools being open bubbles might be most effective if they aligned with those school contacts, and presumably that would also hold for other types of contacts (work, sport, etc.). It may also be worth considering bubbles as an option during second-wave restrictions, but with the important warning that the authors found that their model outcomes were sensitive to higher R values at the point of implementation of the strategy.  

 I was pleased to see that the code associated with this work is all available online and is nicely organised, reasonably documented, and CC0 licensed, making the results reproducible and the work re-usable.  

 I have a few smaller queries around possible typesetting errors:

- In the section ‘Sensitivity Analysis’ the following appears: “1when” - likely a missing space?
- In the section ‘Sensitivity Analysis’ the following appears: “underlying. parametric assumptions” -  likely an extra full-stop?
- I have some confusion around the typesetting or naming of variables in expressions in Table 2:
   
  - In the ‘Bubble’ transmission rate, we see something that looks like τ _*B*_, but I think that this does not appear in Table 1 - does it correspond to *T*_B_ in Table 1?
  - Similarly in the ‘Household’ transmission rate, I see *ϲ*(i) and τ _*H*_ **- do these correspond to *Ϲ*(i) and *T _H_* in Table 1?
  - I think that the susceptibility term defined in Table 1 might not appear elsewhere, though it is mentioned in words - I could of course have missed it! If it does not appear, should it? If not, the authors might consider removing it from Table 1?

Is the work clearly and accurately presented and does it cite the current literature?

Yes

If applicable, is the statistical analysis and its interpretation appropriate?

Yes

Are all the source data underlying the results available to ensure full reproducibility?

Yes

Is the study design appropriate and is the work technically sound?

Yes

Are the conclusions drawn adequately supported by the results?

Yes

Are sufficient details of methods and analysis provided to allow replication by others?

Yes

Reviewer Expertise:

Network modelling of infectious disease, graphs and networks

I confirm that I have read this submission and believe that I have an appropriate level of expertise to confirm that it is of an acceptable scientific standard.

## References

[1] : Antibody prevalence for SARS-CoV-2 in England following first peak of the pandemic: REACT2 study in 100,000 adults. *medRxiv*.2020; 10.1101/2020.08.12.20173690 10.1101/2020.08.12.20173690

[2] : A penalized likelihood approach to estimate within-household contact networks from egocentric data. *J R Stat Soc Ser C Appl Stat*.2013;62(4) : 10.1111/rssc.12011 629-648 10.1111/rssc.12011 23935218PMC3736605

# Response

**Published:** 2021-03-15

**Authors:** Trystan Leng

**Affiliations:** University of Warwick, UK

We thank both reviewers for their considered and detailed reviews of our manuscript. We

 respond to each of the points raised by reviewers below, and have also revised the main body

 of the manuscript where appropriate.

 Reviewer 1:

- Comment:​ I was initially slightly puzzled by the use of directed edges here (given that a single contact could transmit in either direction), but on reflection it makes sense in the context of the subsampling of the matrix to give essentially a transmission network rather than a contact network. The authors may wish to include a sentence or two clarifying this choice.
  Response:​ Thank you for highlighting this. We have now clarified this in the main body of the test, adding in the sentences in bold:
  “Doing so, we retain only the infectious connections between individuals that will lead to an infection. ​ **These sampled probability matrices therefore represent** ***potential*** **transmission networks (though the exact transmission network for a given** **simulation will depend upon who is initially infected). Because these sampled** **matrices refer to transmission events rather than contacts, this matrix may be** **unsymmetric.**”
  And later
  “C2 is obtained by taking the sampled infectious contacts from Scenario 6, then rewiring these sampled contacts.Doing so keeps the number of secondary infections from any individual constant across both scenarios. ​ **As the sampled contact matrices represent** **transmission networks, edges may be either directed or undirected.**​ ”
- Comment: ​ I would also have been interested in seeing an analysis of bubbling value in a setting with a steadily increasing mean-field risk of transmission, as an attempt to model a general opening-up and increase in activity. However, I realise this is beyond the scope of this work, and I note that the authors discuss this in the Discussion, noting the likelihood of a ‘multi-variable exit strategy’ in which bubbles might combine with other
  restriction easements.
  Response: ​We agree that such an analysis is beyond the scope of this current work.
  However, we believe that Figure 5 provides some intuition as to what would happen in such a setting. Figure 5 shows that bubbling scenarios cause an approximately constant increase to ​ R, demonstrating that over the range of mean-field transmissions considered in Figure 5 bubble strategies have a similar impact on R.
- Comment: ​ I wanted to highlight an assumption that I think is important for this model, and that may not apply in all situations: the idea that “We therefore assume that the risk of a household acquiring infection from the community is independent of its number of occupants”. While I think this is well-supported by citations and is appropriate within the setting considered here, I just wanted to highlight that this may not hold true in larger
  households composed of multiple unrelated adults - e.g. in student households. In the REACT-2 reports of antibody prevalence
  (https://www.medrxiv.org/content/10.1101/2020.08.12.20173690v21) after several months of the pandemic in England, individuals in larger households were more likely to have antibodies - of course this could also be potentially explained by a fixed within-household SAR regardless of household size, and the many risk-factors captured within REACT-2 correlated with living in a large household. The authors include a
  violation of this assumption in the sensitivity analyses, and if I have understood correctly it doesn’t qualitatively change the findings.
  Response: Thank you for highlighting this. We have updated our discussion to highlight that the assumption of external infection being independent of household size may not apply to all contexts, and have included the suggested reference. In our sensitivity analyses we consider the situation where a household’s probability of acquiring infection from the community scales with household size. This does not qualitatively change our findings, but does impact on the order of which bubbling scenario results in the smallest increase in R (for example, Scenario 3 now results in the smallest increase in ​ R ​ , as
  Scenario 3 focuses on single-occupancy households). In reality, the risk of external infection to a household is probably between these two extremes, and may vary from household to household. We have also included a fixed within-household SAR regardless of household size (i.e. density dependent transmission) in our sensitivity
  analysis. Again, this does not qualitatively change our results, but does impact the ordering of scenarios. (Added text in bold below:)
**“As in any epidemiological modelling study, we must make some assumptions surrounding transmission. Firstly, we assumed that transmission across close contacts was frequency dependent, informed by previous studies that indicate the probability of infection across two specific members of the same household decreases with household size for COVID-19 (and other communicable diseases**
** like influenza). However, there remains uncertainty surrounding the nature of close-contact transmission for COVID-19, and the assumption of frequency dependence may not accurately capture transmission across all settings.** **Secondly, we assumed that the risk of a household acquiring infection from the community is independent of its number of occupants. While this assumption may** **be appropriate for some households (e.g. families where one adult leaves the** **household to do shopping), it may not hold true in other contexts (e.g. student** **households comprising of largely independent individuals).. Because of this we tested the sensitivity of our results to these assumptions by alternatively** **considering close contact transmission as density dependent, and allowing the** **risk of household infection to increase with household size. These alternative assumptions do not qualitatively change our findings, but do change the ordering** **of the risk of social bubble strategies. In particular, under either of these** **alternative assumptions, social bubble strategies targeting single occupancy** **households result in a lower increase in R than strategies targeting families with** **children. Reports on the antibody prevalence in England found household size associated with antibody prevalence, suggesting that household size may play a** **role in the probability of acquiring infection; a more detailed understanding of the nature of close contact and community transmission may help inform more** **precise evaluations of the effectiveness of social bubbles.”**
- Comment:​ I also found it interesting that the authors chose to have the mean-field transmission depend on an individual’s household size, but not on bubble size would we expect bubble interactions to decrease interactions in the community that would contribute to the general spread modelled as mean-field here? Am I correct in thinking that moderating the mean-field transmission including bubble size would have increased the efficacy of bubbles?
  Response: ​We chose to focus on scenarios where implementing bubble strategies would strictly increase transmission. If a household’s mean-field transmission depends on the size of their bubble, the introduction of a social bubble strategy may in some instances decrease transmission (the increase in within-bubble transmission may be less than the decrease in community infection). You are correct in saying that this would have increased the efficacy of bubbles, and we have now noted this in the discussion (added text in bold below):
**“We do not consider the risk of community transmission depending on bubble**
** size. However, if bubbles were to act as cohesive units, and as a consequence**
** reduce their interaction with the community, this may further increase the**
** effectiveness of social bubble strategies.”**
- Comment: ​ The authors note that the epidemic risk was sensitive to a much higher than observed secondary attack rate within a household - I wanted to note that this parameter is still somewhat uncertain, though their estimate is very reasonably based in observation. Again (and this does not detract from the application of this work to a general population), this may differ in very unusual e.g. large young-adult student households due to differing network effects within the household (for example, as described in https://rss.onlinelibrary.wiley.com/doi/10.1111/rssc.120112)
  Response: Thank you for highlighting this. We have updated our discussion to highlight
  the uncertainty that remains in determining this parameter, and the heterogeneity in attack rates that may occur in different settings. (Added text in bold below):
  “ **There remains some uncertainty surrounding the household attack rate of**
** COVID-19, with ​**high household attack rates observed in some instances. ​However,​ our base case assumptions are in line with an increasingly consistent picture emerging in the contemporary academic literature. Also, superspreading events have been raised as apotentially important source for sustained transmission of SARS-CoV-2, which would
  further imply a rather low secondary household attack rate in most instances. ​ **However,**
** household attack rates may vary between different types of household, and may be larger for some households with unusual network structures, such as large student households.”**
- Comment: ​ There are many possible extensions to this work that could take it beyond the emerging-from lockdown scenario - the authors mention that in the case of schools being open bubbles might be most effective if they aligned with those school contacts, and presumably that would also hold for other types of contacts (work, sport, etc.). It may also be worth considering bubbles as an option during second-wave restrictions, but with
  the important warning that the authors found that their model outcomes were sensitive to higher R values at the point of implementation of the strategy.
  Response: ​ We have included in the discussion that social bubbles aligned with other types of contacts, such as sport or workplace, would likely improve the efficacy of any bubbling strategy (Added text in bold below):
  “As lockdown measures are eased and schools are gradually re-opened forming social bubbles that largely overlap with societal contacts one (e.g. forming social clusters with families that have children going to the same class) is likely further reducing the additional epidemic risk from social bubbles. ​ **By the same token, as lockdown eases,**
** social bubbles aligned with other types of contacts, such workplace contacts or sport team contacts, would likely improve the efficacy of any bubbling strategy.​** ”

Minor comments:

- Comment: ​In the section ‘Sensitivity Analysis’ the following appears: “1when” - likely a missing space?
  Response: ​ This has now been amended.
- Comment: ​ In the section ‘Sensitivity Analysis’ the following appears: “underlying. parametric assumptions” - likely an extra full-stop?
  Response: ​This has now been amended.
- Comment: ​ In the ‘Bubble’ transmission rate, we see something that looks like τ​ B​ , but I think that this does not appear in Table 1 - does it correspond to T​ B​ in Table 1? Similarly in the ‘Household’ transmission rate, I see c(i) and τ​ H​ - do these correspond to C(i) and T​ H​ in Table 1?
  Response: ​We apologise for the typesetting errors in notation. The correct notation for household and transmission rates is τ​ H​ and τ​ B​ , and the correct notation the susceptibility scaling factor is C(i). Tables have been amended and now notation is applied consistently throughout the paper.
- Comment: ​ I think that the susceptibility term defined in Table 1 might not appear elsewhere, though it is mentioned in words - I could of course have missed it! If it does not appear, should it? If not, the authors might consider removing it from Table 1?
  Response: ​ The susceptibility term appears in the equation for R(g)
